# Wu Zi Yan Zong Wan enhances sperm quality in boars by regulating spermatogenesis-related pathways and gut microbiota

**DOI:** 10.3389/fvets.2026.1748849

**Published:** 2026-03-05

**Authors:** Weijie Lv, Xinglin Gao, Haoxuan Zhao, Menghui Cao, Lizhen Zhou, Shuhong Chai, Yun Guan, Jiale Lian, Qian Qu, Shining Guo

**Affiliations:** College of Veterinary Medicine, South China Agricultural University, Guangzhou, China

**Keywords:** boar, gut microbiota, sperm quality, traditional Chinese medicine, Wu Zi Yan Zong Wan

## Abstract

*Wu Zi Yan Zong Wan* (*WZYZW*) is a well-known Traditional Chinese Medicine formula used to treat low sperm quality, although its underlying therapeutic mechanism remains incompletely elucidated. This study aimed to elucidate the therapeutic mechanism of *WZYZW* for low sperm quality in breeding boars and to evaluate its potential for extending their reproductive lifespan. Boars with low sperm quality were pre-screened using the CASA system and *WZYZW* was added to the diet as an intervention. Sperm viability, ejaculate volume, sperm density, FSH, LH, testosterone T, Ca^2+^, Zn^2+^, T-AOC, GSH-Px, SOD and Giemsa staining were evaluated after 8 weeks to evaluate the therapeutic effect of *WZYZW* on boars with low sperm quality. Furthermore, an investigation was conducted into the potential mechanisms of *WZYZW* for treating low sperm quality in boars. This investigation utilized quantitative polymerase chain reaction (qPCR), 16S RNA sequencing, and thermal correlation analysis. *WZYZW* significantly restored sperm motility, ejaculate volume, and sperm density in boars with initial poor semen quality. The treatment also effectively reduced sperm deformities and increased the levels of FSH, testosterone (T), fructose, Ca^2+^, Zn^2+^, total antioxidant capacity (T-AOC), glutathione peroxidase (GSH-Px), and superoxide dismutase (SOD). At the molecular level, *WZYZW* altered chromatin structure and the expression of adhesion protein family genes, potentially via the cAMP-AKT pathway. Furthermore, 16S rRNA sequencing revealed that *WZYZW* significantly suppressed the relative abundance of the phyla *Tenericutes* and *Proteobacteria*. Correlation analysis suggested that the abundance of these gut microbiota taxa may be associated with signaling pathways regulating cell growth and death. Our results demonstrate that *WZYZW* is a promising therapeutic agent for low sperm quality in boars, which acts by modulating spermatogenesis-related gene expression and reshaping the gut microbiota, thereby extending the reproductive lifespan of breeding boars.

## Graphical abstract

**Figure:**
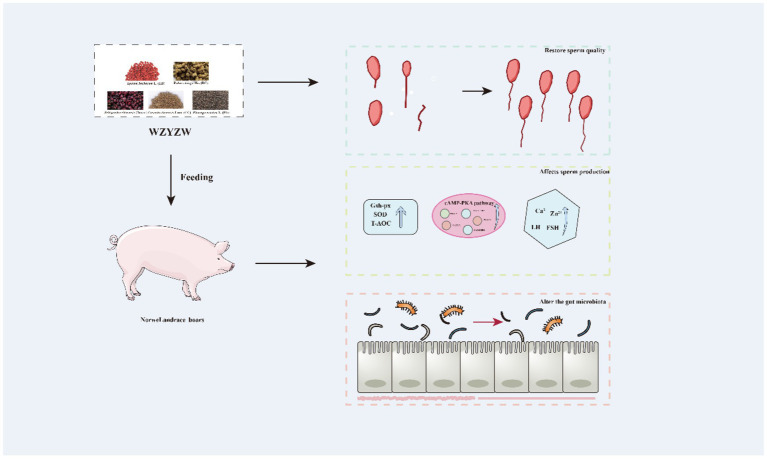

## 1 Introduction

Pigs are a vital source of protein for people worldwide, and enhancing pig breeding capacity is essential for sustainable and economically efficient pork production (1). Artificial insemination plays a crucial role in pig breeding; thus, the quality of the boar’s semen is closely linked to the success of artificial insemination and the growth performance of future fattening pigs (2). At present, the primary factors improving semen quality in boars include feed supplementation with copper, zinc, and molybdenum, medications that promote the production of relevant sex hormones, and treatment with Chinese herbal medicine(CHM) (3, 4).

There is evidence that herbs improve sperm quality mainly in terms of ejaculate volume, sperm motility, and reduce the rate of sperm abnormalities (5, 6). Various herbal ingredients and formulas derived from global traditional medicine systems, including Traditional Chinese Medicine (e.g., *Qilin Pill*, *W Z Y Z W*), have been investigated for their potential effects on sperm motility (7–9).

*WZYZW* is a traditional formula derived from the “Xuanjielu,” which comprises *Lycium barbarum* L. (LB), *Cuscuta chinensis* Lam. (CC), *Rubus chingii* Hu. (RC), *Schizandra chinensis* (Turcz.) Baill. (SC), and *Plantago asiatica* L. (PA) (10). In clinical practice, it is widely used for symptoms such as kidney deficiency with low semen content, phallic fistula and premature ejaculation, spermatorrhea, and seminal coldness (He et al., 2012). However, the underlying principle of *WZYZW* in treating these symptoms remains unclear.

However, the underlying principle of *WZYZW* in treating these symptoms remains unclear. Emerging evidence points to the gut–testicle axis as a potential mediator for herbal efficacy, where gut microbiota can influence sperm quality via energy metabolism, spermatogenesis, and hormone production. In addition, recent studies have found that gut microbiota transplantation can effectively improve sperm quality in type 2 diabetes (11, 12). Therefore, gut microbiota may be an effective way to improve sperm quality.

In this paper, based on the previous studies (13), breeding boars with low sperm motility and high malformation rate were screened as the model group. The direct effect relationship of *WZYZW* on the sperm quality of breeding boars was elucidated through the markers related to the reproductive ability of boars. In addition, by using 16 s rRNA gut microbiota sequencing analysis, the related effects of *WZYZW* on gut microbiota were investigated, which provides a certain research basis for future research on the gut microbiota-reproductive axis.

## 2 Materials and methods

### 2.1 Animal treatment and experimental design

The study involved 36 adult Landrace boars (12–14 months old, weighing 200–230 kg) at the Guangdong Gu Yue Science and Technology Co., Ltd. breeding station. The boars were classified into two categories: good semen quality (motility ≥85%, deformity <20%) and low semen quality (motility ≤60%, deformity ≥30%). They were divided into four groups: Con (control), Abn (sperm abnormalities), L-WZ (low-dose *WZYZW* compound, 0.05 g/kg), and H-WZ (high-dose *WZYZW*, 0.15 g/kg). Each group consisted of nine boars fed a commercial corn-soybean meal diet for 16 weeks, with controlled intake and environmental conditions (20° ± 3C, 60–75% humidity). The experiment was approved by the Ethics Committee of South China Agricultural University (No. 2024F361).

### 2.2 Collection and preservation of semen samples

At weeks 0, 4, 8, 12, and 16, semen samples were collected from all boars using standardized operating procedures to ensure consistency across time points. Samples were obtained by trained personnel wearing sterile gloves using the hand-held semen collection method. The mid-stream portion of the ejaculate, rich in sperm, was primarily collected. Samples were immediately transported to the laboratory via delivery tubes. To minimize pre-analytical variability, semen from each boar was gently mixed, placed in thermostatic containers maintained at 37 °C, and aliquoted into 5-milliliter sterile tubes within 30 min of collection. All samples were subsequently stored at −80 °C for subsequent batch analysis.

### 2.3 Ejaculation volume

After weighing the boar’s ejaculate volume using a balance, the ejaculate volume was calculated according to the specific gravity of the boar’s semen.

### 2.4 Determination of sperm motility, sperm density and malformation rate in boars

Measurement of sperm motility, sperm density and malformation rate using the Computer Assistant Semen Analysis (CASA) system. According to the World Health Organization guidelines, 15 μL of semen was extracted into a four-chambered sperm counting plate for the CASA system, and then placed on a preheated carrier plate at 37 °C to calculate the semen indexes of the boars using the CASA automated analysis system. To eliminate individual differences, the valid values of five different fields of view were recorded and averaged to obtain the sperm motility, sperm density and sperm malformation rate.

### 2.5 Wright-Giemsa stain

Briefly, the staining steps are preparation of smear, fixation, and elution, followed by subsequent steps as per instructions. Rachel-Giemsa staining solution was purchased from Fuzhou FeiJing Biotechnology Company Limited (Item No.: PH1793).

### 2.6 Detection of plasma and seminal plasma sex hormones and seminal plasma antioxidant indices in breeding boars

Accurately measure boar plasma, seminal plasma sex hormones and seminal plasma antioxidants according to the steps in the ELISA kit instructions. ELISA kits were purchased from Shanghai Enzyme-linked Biotechnology Co. FSH kit, Item No. ml002326; Testosterone kit, Item No. ml002339; LH kit, Item No.ml104023; SOD kit, Item No. ml002400; GSH-PX assay kit, Item No. ml095262; T-AOC assay kit, Item No. ml094998.

### 2.7 Detection of trace elements and fructose content in seminal plasma

Seminal plasma samples were deionized and diluted 3-fold for Ca^2+^ and fructose content prior to the test, and the plasma used for Zn^2+^ content was left untreated. Calcium (Ca^2+^) Test Kit, Item No. C004-2-1; Zinc (Zn^2+^) Determination Kit, Item No. E011-1-1; Fructose Determination Kit, Item No. A085-1-1; purchased from Nanjing Jianjian Bioengineering Research Institute Effective Company.

### 2.8 Quantitative real-time polymerase chain reaction (qRT-PCR) analysis

Real-time quantitative PCR (qRT-PCR) analysis using previous methods. Rna extraction and expression calculations were consistent with our previous studies (14). During the extraction process, to enhance the recovery of sperm RNA, we added *β*-mercaptoethanol to the Trizol solution (15). Primers were shown in the (Supplementary Table S1).

### 2.9 Composition of WZYZW

WZYZW comprises *Lycium barbarum* L. (LB), *Cuscuta chinensis* Lam. (CC), *Rubus chingii* Hu. (RC), *Schizandra chinensis* (Turcz.) Baill. (SC), and *Plantago asiatica* L. (PA). For further details, please refer to the Supplementary Table S2.

### 2.10 16 s rRNA sequencing

To analyze the fecal microbial structural composition of boars, 16 s rDNA sequencing was used to examine boar fecal samples. The total DNA was extracted using DNeasy PowerSoil Kit, followed by PCR amplification, product recovery, preparation of sequencing library, and final sequencing. DNeasy PowerSoil Kit, Beijing Unikon Biotechnology Co., Ltd.; Pfu high-fidelity DNA polymerase, Beijing QuanShiJin Biotechnology Co., Ltd.; Nucleic acid purification beads BECKMAN AMPure XP BECKMAN AMPure XP Beads, courtesy of Shanghai Parseno Bio-technology Co.

### 2.11 Bioinformatics

Sankey diagrams were drawn using the Sangrbox online tool, while correlation heat maps were drawn using Shanghai Parseno Bio-technology Co (16).

### 2.12 Statistical analysis

Data are expressed as the mean ± standard deviation (SD) from at least three independent experiments. Statistical analyses were performed using GraphPad Prism 8.0 software (GraphPad Software, La Jolla, CA, US). For comparisons at a single time point or endpoint measurements, mean differences were assessed using Student’s t-test or one-way analysis of variance (ANOVA), followed by Tukey’s *post hoc* test when necessary. For parameters measured repeatedly over time (e.g., semen quality parameters at weeks 0, 4, 8, 12, and 16), repeated-measures ANOVA (RM-ANOVA) was used to assess the within-group effect of time, the between-group effect of treatment, and the time × treatment interaction. When significant interactions were detected, post-hoc comparisons were performed at each time point using one-way ANOVA combined with Tukey’s test. A *p*-value < 0.05 was considered statistically significant. Graphing with GraphPad Prism 8 and STAMP Software.

## 3 Results

### 3.1 Effect of *WZYZW* on the quality of porcine spermatozoa

**Figure 1:**
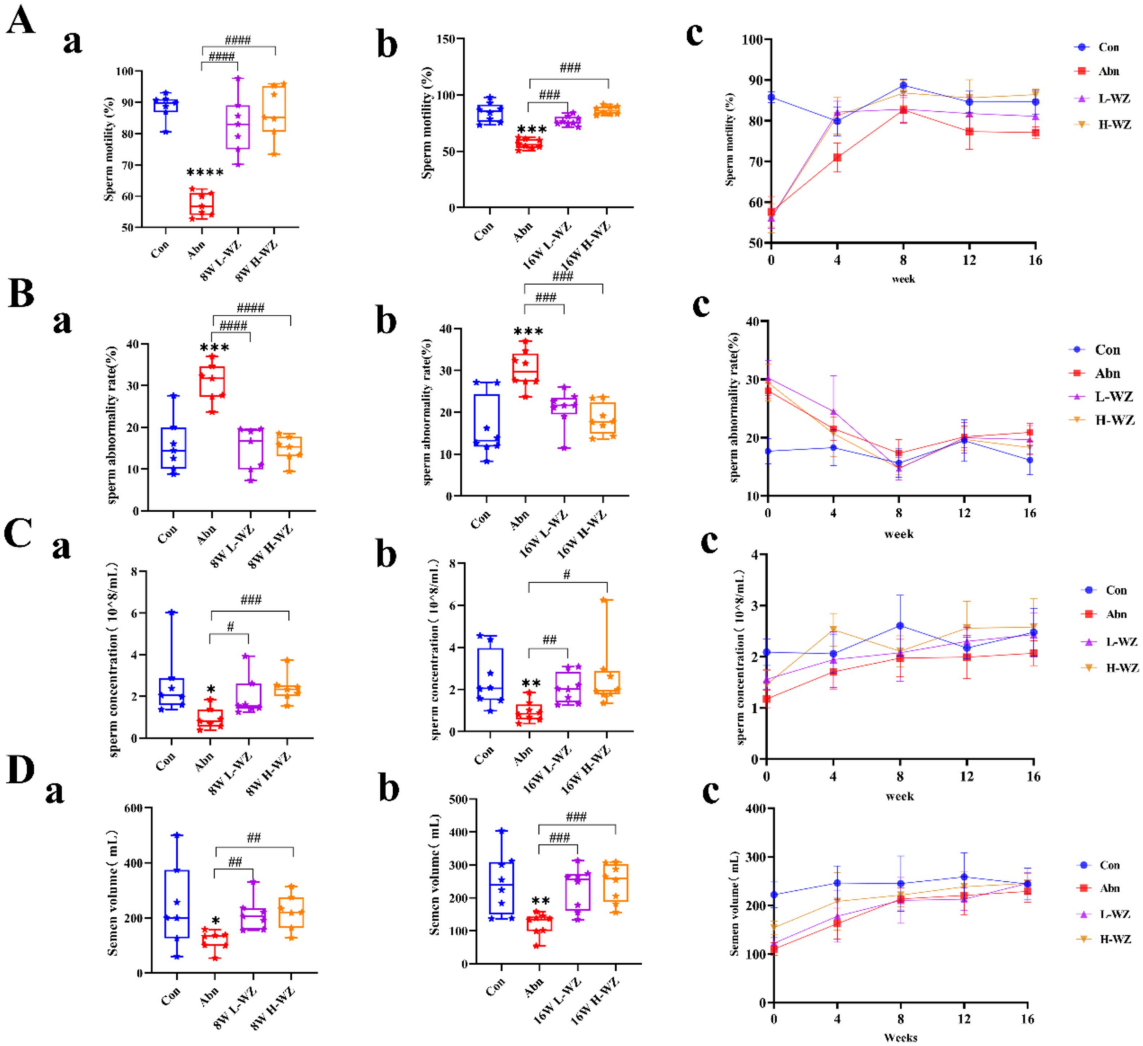
**(A)** Sperm motility. **(B)** Sperm abnormality rate. **(C)** Sperm concentration. **(D)** Semen volume. “*” indicated significant difference compared to the corresponding control (*p < 0.05, **p < 0.01 and ***p < 0.001). “#” indicated statistically significant difference between corresponding group (#*p* < 0.05, ##*p* < 0.01, and ###*p* < 0.001).

Figures 1Aa–c show the effect of *WZYZW* on boar sperm motility. The results showed that *WZYZW* significantly increased sperm motility from 8 weeks to 16 weeks (*p* < 0.001). Figures 1Ba–c shows that *WZYZW* significantly reduced sperm abnormality rate at 8–16 weeks (*p* < 0.001). Furthermore, Figures 1Ca–c shows that after 8–16 weeks of *WZYZW* feeding, it effectively restored the sperm count of boars (*p* < 0.05, *p* < 0.01, *p* < 0.001). Figures 1Da–c reveals the effect of *WZYZW* on the ejaculate volume of boars, with similar results from 8 to 16 weeks, where *WZYZW* significantly increased the ejaculate volume (*p* < 0.05, *p* < 0.01, *p* < 0.001).

### 3.2 Effect of *WZYZW* on sperm morphology

**Figure 2:**
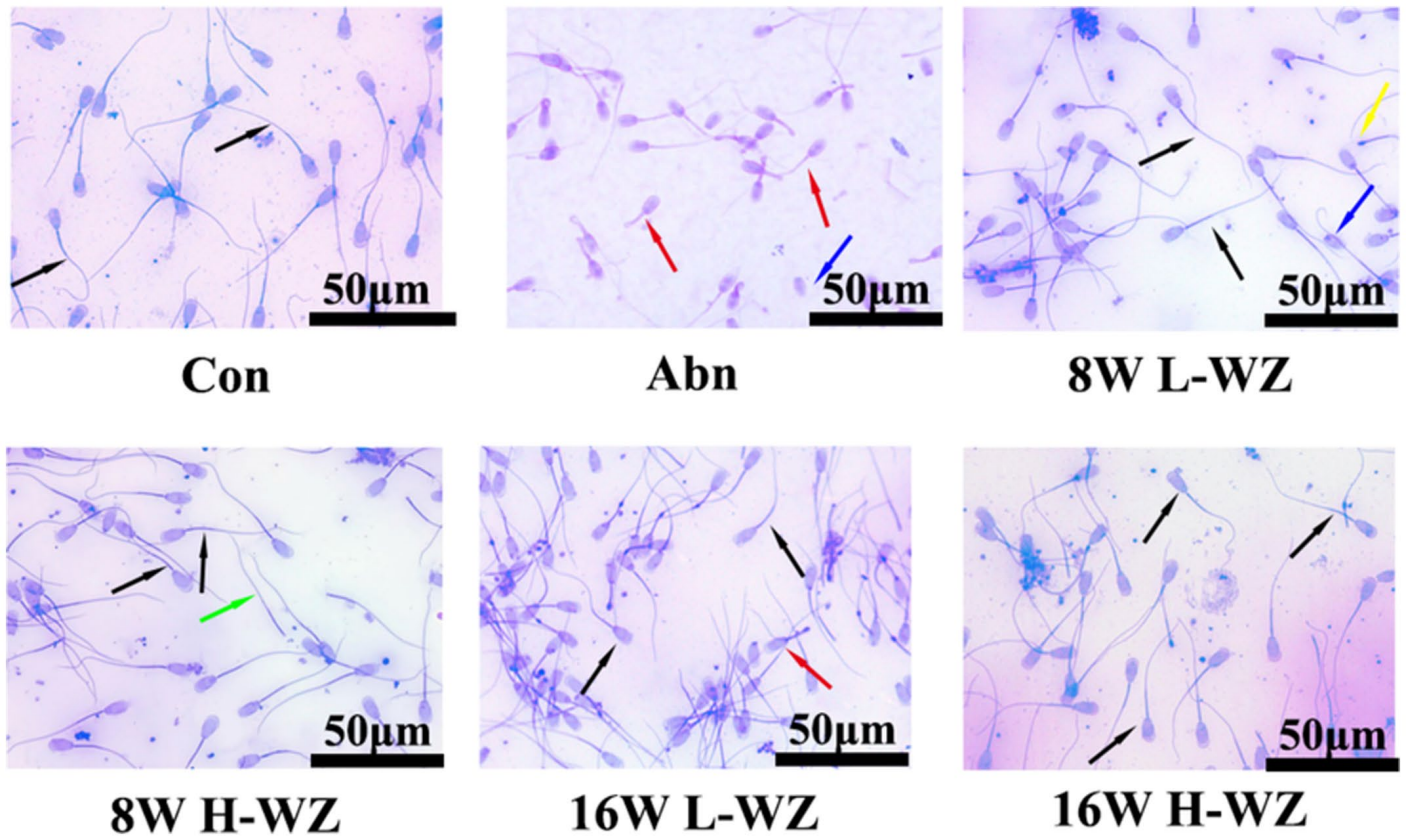
Sperm Wright-Giemsa staining (Scale bar: 50 μm). Black arrowheads indicate normal mature sperm morphology, red arrowheads indicate immature sperm morphology, yellow arrowheads indicate sperm with abnormal acrosome development, blue arrowheads indicate sperm with absent caudal flagellum, and green arrowheads indicate sperm with absent acrosome.

Figure 2 shows the effect of *WZYZW* on sperm morphology, which effectively restored the abnormal development of acrosome and the absence of flagellum and acrosome, and promoted the normal development of spermatozoa.

### 3.3 Effects of *WZYZW* on sperm sex hormones, trace element and antioxidant levels

**Figure 3:**
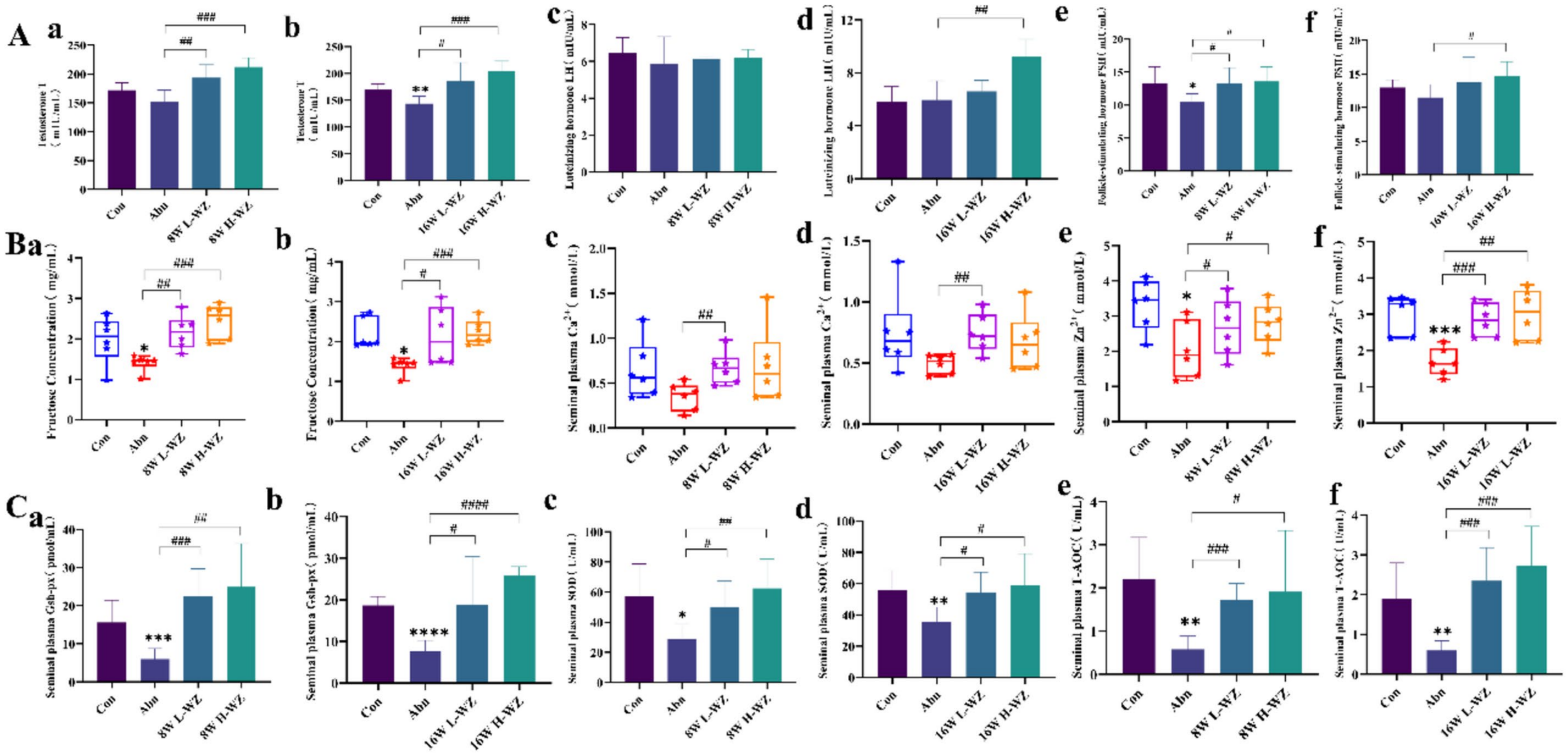
**(A, a,b)** Sperm T content. **(A, c,d)** Sperm LH content. **(A, e,f)** Sperm FSH content. **(B, a,b)** Sperm fructose content. **(B, c,d)** Sperm Ca^2+^ content. **(B, e,f)** Sperm Zn^2+^ content. **(C, a,b)** Sperm GSH-Px content. **(C, c,d)** Sperm SOD content. **(C, e,f)** Sperm T-AOC content. “*” indicated significant difference compared to the corresponding control (*p < 0.05, **p < 0.01 and ***p < 0.001). “#” indicated statistically significant difference between corresponding group (#*p* < 0.05, ##*p* < 0.01, and ###*p* < 0.001).

The effect of *WZYZW* on sperm sex hormone content is presented in Figure 3A. In Figure 3A panels a,b, the effect of *WZYZW* on testosterone T content is shown, with results indicating that at 8 weeks, similar to the findings at 16 weeks, *WZYZW* effectively restored testosterone T content in the Abn group (*p* < 0.05, *p* < 0.01, *p* < 0.001). Figure 3A panels c,d illustrate the effect of *WZYZW* on luteinizing hormone content, revealing that after 8 weeks of treatment, the effect of *WZYZW* on sperm luteinizing hormone content was not significant (*p* > 0.05). Only the 16-week H-WZ group exhibited a significant effect compared to the Abn group (*p* < 0.01). Figure 3A panels e,f demonstrate the impact of *WZYZW* on follicle-stimulating hormone content in spermatozoa, where results show that after 8 weeks of treatment, all *WZYZW* groups significantly restored the expression of follicle-stimulating hormone in the Abn group (*p* < 0.05, *p* < 0.01), while only the H-WZ group showed a significant restoration in follicle-stimulating hormone expression in the Abn group at 16 weeks (*p* < 0.01).

After 16 weeks of premixing, the effect of *WZYZW* on the expression of relevant sex hormones in blood was similar to that at 8 weeks, as detailed in Supplementary Figure S1.

The effects of *WZYZW* on the content of relevant trace elements in spermatozoa are shown in Figure 3B. Specifically, Figures 3Ba,b illustrates the impact of *WZYZW* on fructose levels, showing that the results after 8 weeks were similar to those after 16 weeks, and *WZYZW* effectively restored fructose content in the Abn group (*p* < 0.05, *p* < 0.01, *p* < 0.001). Furthermore, Figures 3Bc,d demonstrates *WZYZW*’s effects on the Ca^2+^ content, with significant differences mainly observed in the L-WZ group at both 8 and 16 weeks compared to the Abn group (*p* < 0.01). Finally, Figures 3Be,f reveals the effect of *WZYZW* on Zn^2+^ levels in spermatozoa, indicating similar results at 8 and 16 weeks, with *WZYZW* effectively increasing Zn^2+^ content in the Abn group (*p* < 0.05, *p* < 0.01, *p* < 0.001).

Figure 3C shows the effect of *WZYZW* on antioxidant indices in spermatozoa. Specifically, Figure 3C illustrates the effects of *WZYZW* on the content of Gsh-px, SOD and T-AOC, respectively, and the results showed that *WZYZW* effectively restored the content of Gsh-px, SOD and T-AOC in the Abn group (*p* < 0.05, *p* < 0.01, *p* < 0.001).

### 3.4 Effect of *WZYZW* on spermatogenesis-related genes and pathways after 8 weeks of premixing

**Figure 4:**
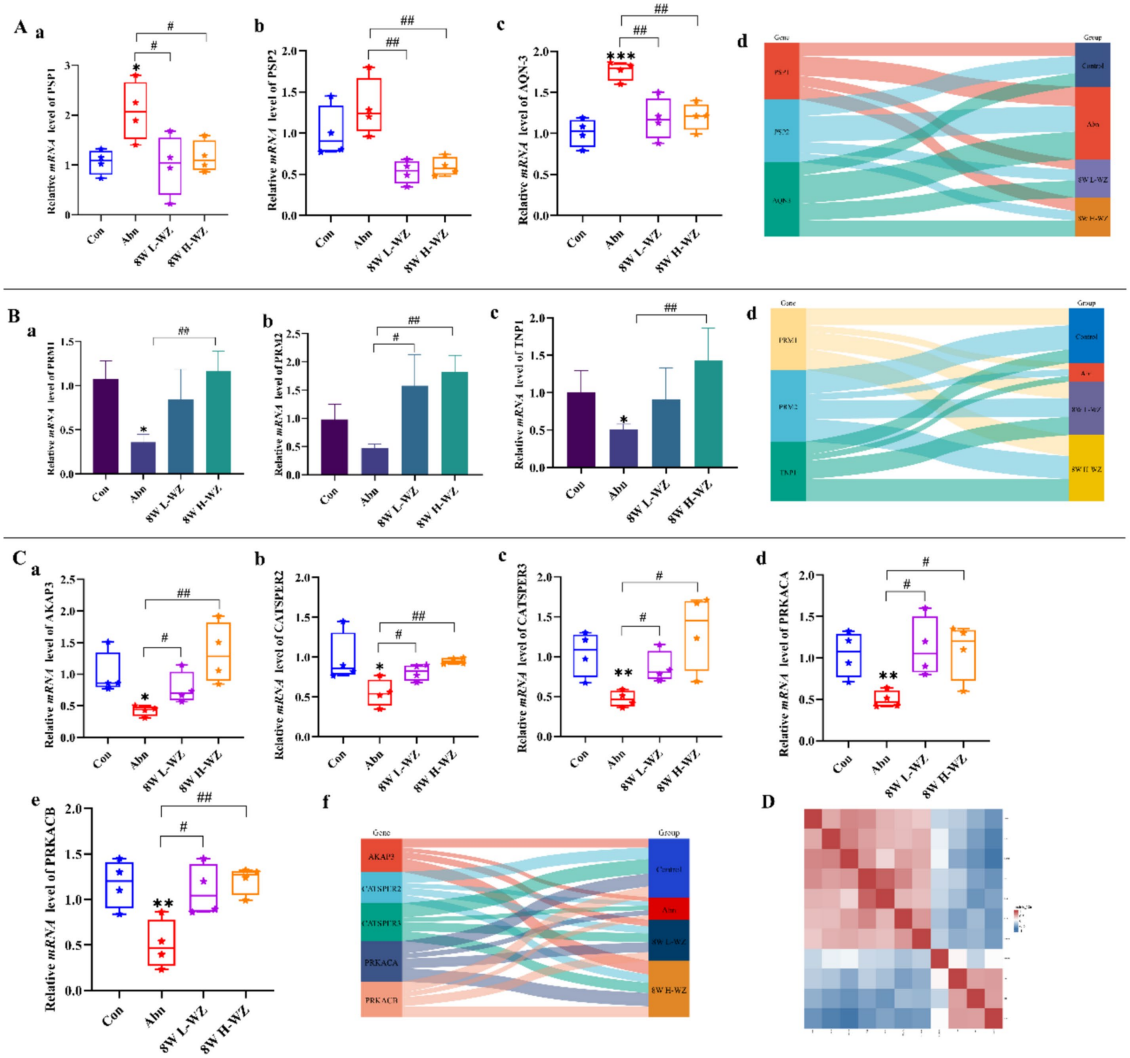
**(A)** Sperm adhesion protein family mRNA expression **(A–D)**. **(B)** Sperm chromatin formation-associated mRNA expression **(A–D)**. **(C)** Sperm cAMP-PKA pathway-related mRNA expression **(A–F)**. **(D)** Heatmap of mRNA correlation. “*” indicated significant difference compared to the corresponding control (*p < 0.05, **p < 0.01, and ***p < 0.001). “#” indicated statistically significant difference between corresponding group (#*p* < 0.05, ##*p* < 0.01, and ###*p* < 0.001).

The effects of *WZYZW* on the expression of mRNAs related to the sperm adhesion protein family are shown in Figures 4Aa–d. Compared with the Abn group, *WZYZW* significantly decreased the mRNA expression of the adhesion proteins FSP1, FSP2 and AQN-3 in sperm (*p* < 0.05, *p* < 0.01).

The effects of *WZYZW* on chromatin formation-related mRNA expression in spermatozoa are shown in Figures 4Ba–d. H-WZ significantly increased the mRNA expression of chromatin formation-related genes PRM1, PRM2 and TNP1 in spermatozoa compared to the Abn group (*p* < 0.01, *p* < 0.001).

The effects of *WZYZW* on the mRNA expression of the cAMP-PKA pathway in spermatozoa are shown in Figures 4Ca–f. Compared with the Abn group, both L-WZ and H-WZ significantly increased the expression of mRNA related to the cAMP-PKA pathway in spermatozoa (*p* < 0.05, *p* < 0.01, *p* < 0.001).

Figure 4D shows a correlation heat map of the effect of *WZYZW* on spermatogenesis-related genes and pathways after 8 weeks of premixing, with positive correlations in red and negative correlations in blue.

After 16 weeks of premixing, the effect of *WZYZW* on mRNA expression of spermatogenesis-associated genes and pathways was similar to that at 8 weeks, as detailed in Supplementary Figure S2.

### 3.5 Effect of *WZYZW* on gut microbiota

**Figure 5:**
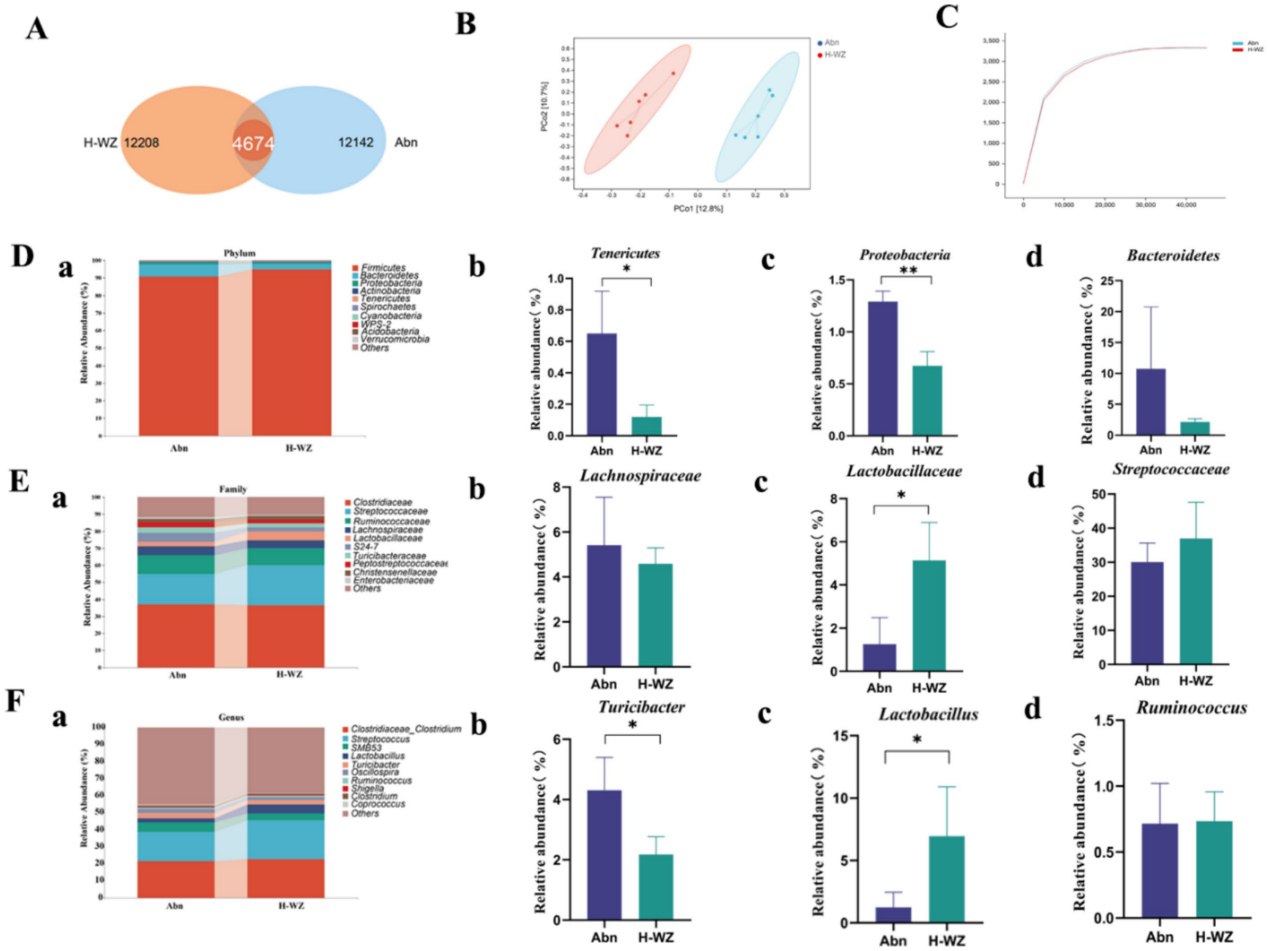
**(A)** The Venn diagram analysis of OTUs. **(B)** PCoA plot analysis from each sample. **(C)** Species sparseness curve. **(D)** Microbial composition at the phylum level **(A–D)**. **(E)** Microbial composition at the family level **(A–D)**. **(F)** Microbial composition at the genus level **(A–D)**. “*” indicated significant difference compared to the corresponding control (*p < 0.05, **p < 0.01 and ***p < 0.001). “#” indicated statistically significant difference between corresponding group (#*p* < 0.05, ##*p* < 0.01, and ###*p* < 0.001).

To investigate the effect of *WZYZW* on the gut microbiota, we amplified and sequenced the V3 + V4 regions of ileal contents 16S rDNA. Through Venn evaluation of OTUs, we studied the overlap and resemblance of OUT composition between H-WZ and Abn groups. As shown in Figure 5A, the number of OUTs unique to boars in the H-WZ group was 12,208, the number of OUTs unique to the Abn group was 12,142, and the number of OUTs common to both was 4,674.

The PCoA results are shown in Figure 5B, where the H-WZ and Abn groups are farther apart, suggesting a larger gap between the two groups of samples.

The trend of species sparsity curves was relatively flat and saturated, indicating that the amount and depth of sequence met the requirements for subsequent analyses (Figure 5C).

The distinguishing characteristics of each group of microorganisms in the phylum, family, genus, and species are illustrated in Figures 5D–F. Figure 5D shows that at the phylum level, H-WZ significantly reduced the abundance of *Tenericutes* with *Proteobacteria* compared to the Abn group (*p* < 0.05). It also reduced the abundance of *Bacteroidetes*, but not significantly (*p* > 0.05). As shown in Figure 5E, at the family level, the H-WZ group significantly increased the abundance of *Lactobacillaceae* compared with the Abn group (*p* < 0.05) while decreasing the abundance of *Lachnospiraceae* and increasing the abundance of *Streptococcaceae*, but none of them were significant (*p* > 0.05). As shown in Figure 5F, at the genus level, the H-WZ group significantly decreased the abundance of *Turicibacter* (*p* < 0.05), significantly increased the abundance of *Lactobacillus* (*p* < 0.05), and simultaneously decreased the abundance of *Ruminococcus* compared to the Abn group, but not significantly (*p* > 0.05).

### 3.6 *WZYZW* correlation analysis of the porcine microbiota with physiological indicators and functional pathways

**Figure 6:**
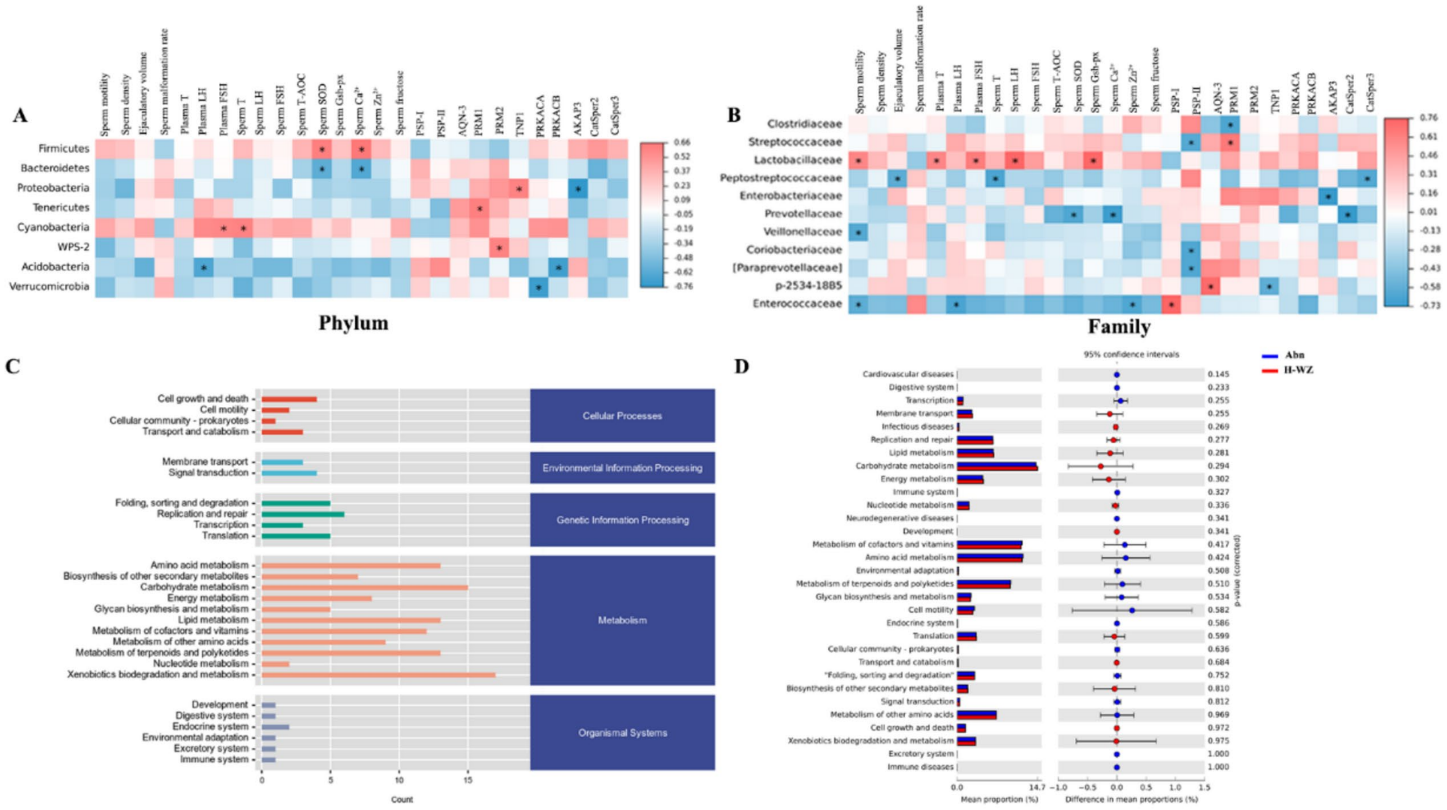
**(A)** Correlation analysis of the phylum of boar microbiota level and blood and semen indicators. **(B)** Correlation analysis of the family of boar microbiota level and blood and semen indicators. **(C)** Boar fecal samples predicted the abundance of KEGG secondary functional pathways. **(D)** Statistical analysis of the significance of secondary KEGG abundance pathway in boar fecal samples.

Based on Spearman’s analysis, correlation heat maps of boar gut microbiota with blood and semen indices were plotted. As shown in Figure 6A, at the phylum level, Firmicutes showed significant positive correlation (*p* < 0.05) with seminal plasma SOD, seminal plasma Ca^2+^, etc., while Bacteroidetes showed significant negative correlation (*p* < 0.05) with seminal plasma SOD, seminal plasma Ca^2+^, etc., Proteobacteria showed a significant positive correlation with sperm TNP1 gene (*p* < 0.05) and AKAP3 gene (*p* < 0.05). Cyanobacteria showed a significant positive correlation with plasma FSH and semen T (*p* < 0.05); Acidobacteria showed negative correlation and significant difference with plasma LH and sperm PRKACB gene (*p* < 0.05).

As shown in Figure 6B, at the genus level, *Lactobacillus* showed significant positive associations with sperm motility, plasma T, plasma FSH, seminal plasma LH, and seminal plasma Gsh-px (*p* < 0.05). *Streptococcus* was negatively associated with the sperm PSP2 gene (*p* < 0.05) and positively associated with the PRM1 gene (*p* < 0.05). *SMB53* was significantly negatively correlated with plasma T and sperm CatSper2 gene (*p* < 0.05), *Ruminococcus* was significantly negatively correlated with sperm PSP2 gene (*p* < 0.05), *Prevotella* was negatively correlated with sperm plasma Ca^2+^ and sperm CatSper2 gene with significant difference (*p* < 0.05). Dorea was significantly positively correlated with plasma FSH was significantly positively correlated (*p* < 0.05) and negatively correlated (*p* < 0.05) with sperm PSP1 gene.

The average abundance of secondary functional pathways of the KEGG database was counted by the KEGG Bio metabolic Pathway Analysis Database (KEGG Pathway Database) on the fecal samples of boars from Abn group and H-WZ group. As shown in Figure 6C, the secondary functional pathways of the fecal samples from boars of the two groups were mainly concentrated in five relevant directions.

Based on the abundance of data on KEGG metabolic pathways in the two groups, a significant analysis of metabolic pathways was performed using STAMP software. As shown in Figure 6D, compared with the Abn group (shown in blue), the fecal samples of boars in the H-WZ group (shown in red) contained Membrane transport, Replication and repair, Lipid Membrane transport, Replication and repair, Lipid metabolism, Carbohydrate metabolism, Energy metabolism, Development, Nucleotide metabolism, Translation, Biosynthesis of other secondaty metabolites, Cellular metabolites, and Cellular metabolism. Metabolites, Cell growth and death, and Xenobiotics biodegradation and metabolism pathway upregulation. And Cardiovascular diseases, Digestive system, Transcription, Immune system, Neurodegenerative diseases, Metabolism of cofactors and vitamins, Amino acid metabolism, Environmental adaptation, Metabolism of terpenoids and polyketides, Glycan biosynthesis and metabolism, Cell motility, Endocrine system, Cellular community-prokaryotes, Folding, sorting and degradation, Signal transduction, Metabolism of other amino acids, Excretory system Immune diseases pathway abundance was down-regulated, but none of them were significant (*p* > 0.05).

## 4 Discussion

Our study suggests that *WZYZW* can be a comprehensive treatment for low sperm quality in breeding boars, while its potential therapeutic effect may be associated with, among other factors, changes in the gut microbiota (17). To our knowledge, this study represents the novel application of *WZYZW* for treating low sperm quality in breeding boars and the first exploration of its underlying mechanisms. The efficacy observed in this model is consistent with our previous findings in other species, corroborating the therapeutic potential of *WZYZW* (18–20).

Previous studies have preliminarily confirmed the effect of *WZYZW* on the male reproductive system (21, 22). *WZYZW* significantly improved key semen parameters (motility, concentration, ejaculate volume) and reduced sperm deformity in boars with low sperm quality, as visually confirmed by Giemsa staining.

The effects of sex hormones on reproduction are mainly to regulate a series of reproductive processes including egg production, ovulation, spermatogenesis, fertilization, implantation, and pregnancy (23). Testosterone T is highly correlated with male sexual response, and reduced serum testosterone T levels can be found in men suffering from hypogonadism (24). Among the current effective treatments for male erectile dysfunction is testosterone therapy (25, 26). Additionally, luteinizing hormone and follicle-stimulating hormone are highly correlated with androgenic effects on spermatogonia maturation (27, 28). *WZYZW* elevates serum and seminal levels of testosterone (T), luteinizing hormone (LH), and follicle-stimulating hormone (FSH), while simultaneously increasing seminal fructose content. The rise in serum hormone levels indicates systemic improvement in the hypothalamic–pituitary-gonadal (HPG) axis—the primary regulator of spermatogenesis. The concurrent increase in FSH and LH in semen may reflect passive diffusion from the circulatory system or alterations in the local microenvironment. We report these changes in seminal gonadotropins as associated findings accompanying overall endocrine and phenotypic improvements; their precise functional significance warrants future investigation. Since seminal fructose—the primary energy source for sperm produced from blood glucose—is crucial for capacitation and a key fertility marker, its elevation further corroborates the pro-fertility effects of *WZYZW* (29, 30). Calcium is a critical mediator of sperm capacitation, a process driven by conformational changes induced through the interaction of calcium-binding proteins with divalent cations. Consequently, the intracellular calcium content is directly linked to overall sperm quality (31, 32). An optimal zinc concentration is essential for promoting sperm nuclear maturation and ensuring high sperm quality (33, 34).

Antioxidants such as Gsh-px, SOD play an important role in counteracting the damage to the body caused by free radicals (35). *WZYZW* significantly enhanced spermatozoal antioxidant capacity, reflected in the increased activities of GSH-Px, SOD, and T-AOC, which is likely attributed to its active constituents such as quercetin, chlorogenic acid, and schisandrin (20).

Adhesion proteins, predominantly comprising AQN1, AQN3, AWN, PSP1, and PSP2, constitute approximately 90% of the total protein content in boar seminal plasma (36, 37). AQN3 is associated with stabilizing vesicles in the sperm acrosome and is mainly released during capacitation (38). Our results are in agreement with the previous results, which showed that the expression of mRNA for adhesion proteins was negatively correlated with sperm quality in low-producing boars, and *WZYZW* significantly reduced the gene level of sperm adhesion proteins (39). Sperm chromatin formation-associated proteins act mainly by affecting sperm growth and differentiation (40). Our results demonstrated that *WZYZW* effectively restored the mRNA expression of chromatin-associated proteins (PRM1, PRM2, TNP1). Furthermore, in spermatozoa, cAMP-dependent PKA serves as the major downstream effector; upon activation by elevated cAMP, it catalyzes extensive phosphorylation of flagellar proteins, thereby inducing the flagellar motility essential for fertilization and supporting sperm maturation (41–43).

Gut microbiota are crucial mediators at the diet-host interface, regulating nutrient absorption and utilization while also facilitating inter-organ communication. This is evidenced by well-established axes such as the gut-liver, gut-mammary, and gut-testis axes (44–47). Past research findings have repeatedly confirmed the significant relationship between gut microbiota and sperm quality (48, 49). At the phylum level, *Tenericutes* have been suggested to be involved in carbohydrate storage, mutation repair and environmental responses (50). Angiopoietin maintains intestinal microbial homeostasis by balancing *alpha*-*proteobacteria* and *Lachnosporium* sp. (51). At the family level, *Lactobacillaceae* has been heavily studied in the past, and its main function is to influence other gut microbes, which in turn affects butyrate levels and is ultimately related to innate immunity (52). At the genus level, *Turicibacter* has been shown to significantly affect fat metabolism as its primary function (53).

## 5 Conclusion

In summary, our study significantly advances understanding of the gut-reproductive axis by demonstrating that *WZYZW* improves semen quality and modulates key hormonal and antioxidant pathways. This work provides novel insights into the mechanism of action of *WZYZW* and proposes a viable strategy for extending the reproductive lifespan of boars.

## Data Availability

The raw sequence data reported in this paper have been deposited in the Genome Sequence Archive (Genomics, Proteomics & Bioinformatics 2025) in National Genomics Data Center (Nucleic Acids Res 2025), China National Center for Bioinformation/Beijing Institute of Genomics, Chinese Academy of Sciences (GSA: CRA033665) that are publicly accessible at https://ngdc.cncb.ac.cn/gsa (52, 54).
