# Supplementary material for: Wu Zi Yan Zong Wan enhances sperm quality in boars by regulating spermatogenesis-related pathways and gut microbiota
Source: Front Vet Sci. 2026 Mar 5;13:1748849. doi: 10.3389/fvets.2026.1748849 (PMC12999450; doi:10.3389/fvets.2026.1748849)
Supplement: Supplementary file 1 [file Data_Sheet_1.docx]

**Table S1** Primer sequences

| *Gene* | *Forward Primer (5′to 3′)* | *Reverse Primer (5′to 3′)* |
| --- | --- | --- |
| *CatSper2* | *TTTTGCTGTGGCTGGTGTCT* | *GTTCAGCAGGTCCGAGAAGA* |
| *CatSper3* | *TCAGACTTTTTGAGGTCTCGGA* | *TGTAGCCGTCCCTCCAGTAG* |
| *AKAP3* | *GCACCCAACAAAAGCCTGAG* | *GCCGGGAGTCTTATCCGAAG* |
| *PRKACA* | *GAGCAGGAGAGCGTGAAAGAG* | *GCCGAGGGTCTTGATTCGTT* |
| *PRKACB* | *CGAAGCCTGCCACAAGAATG* | *TGGAGGAACCAGTCGTCTCT* |
| *AQN-3* | *CGCTGACGACGAACTGTGTCTG* | *GGTGAGGTTGAGAGGAAGGATTTGC* |
| *PSP-I* | *GGGCCCAAAACTGAATGTG* | *GTGAGGTTGAGAGTGGGTAT* |
| *PSP-II* | *ATCAAGGACACATCGGGAA* | *TTGCCATTCGGACTTTCTG* |
| *TNP1* | *AAGAGAGGTGGCAGCAAAAG* | *TCACAAGTGGGAGCGGTAAT* |
| *PRM1* | *GCAGACGAAGGAGGAGATGTTGTC* | *GAGTGCGGTGGTCTTGCTACTG* |
| *PRM2* | *AGTCCGAGTGAAAGTCCGCAG* | *TGTGGCTCCTGTGTCTGTAGTGG* |

**Table S2** Composition of WZYZW

| Botanical name | Proportion (%) |
| --- | --- |
| Lycium barbarum L. (LB) | 35 |
| Cuscuta chinensis Lam. (CC) | 35 |
| Rubus chingii Hu. (RC) | 17 |
| Schizandra chinensis (Turcz.) Baill. (SC) | 5 |
| Plantago asiatica L. (PA) | 8 |

**Figure S1**

**
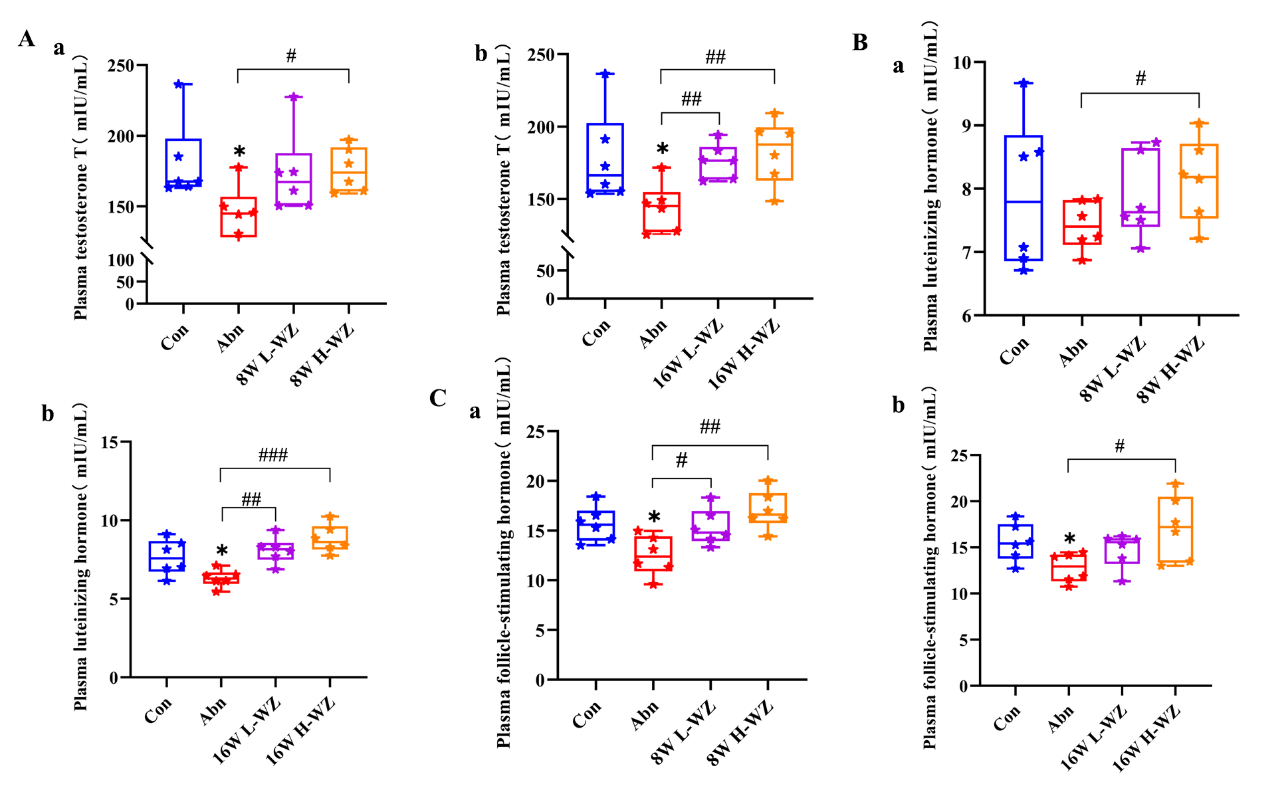
**

**Fig.S1** (A a-b) Plasma T content. (B a-b) Plasma LH content. (Ca-b) Plasma FSH content.“*” indicated significant difference compared to the corresponding control (*P < 0.05, **P < 0.01 and ***P < 0.001). “#” indicated statistically significant difference between corresponding group (#P < 0.05, ##P < 0.01, and ###P < 0.001)

**FigureS2**

**
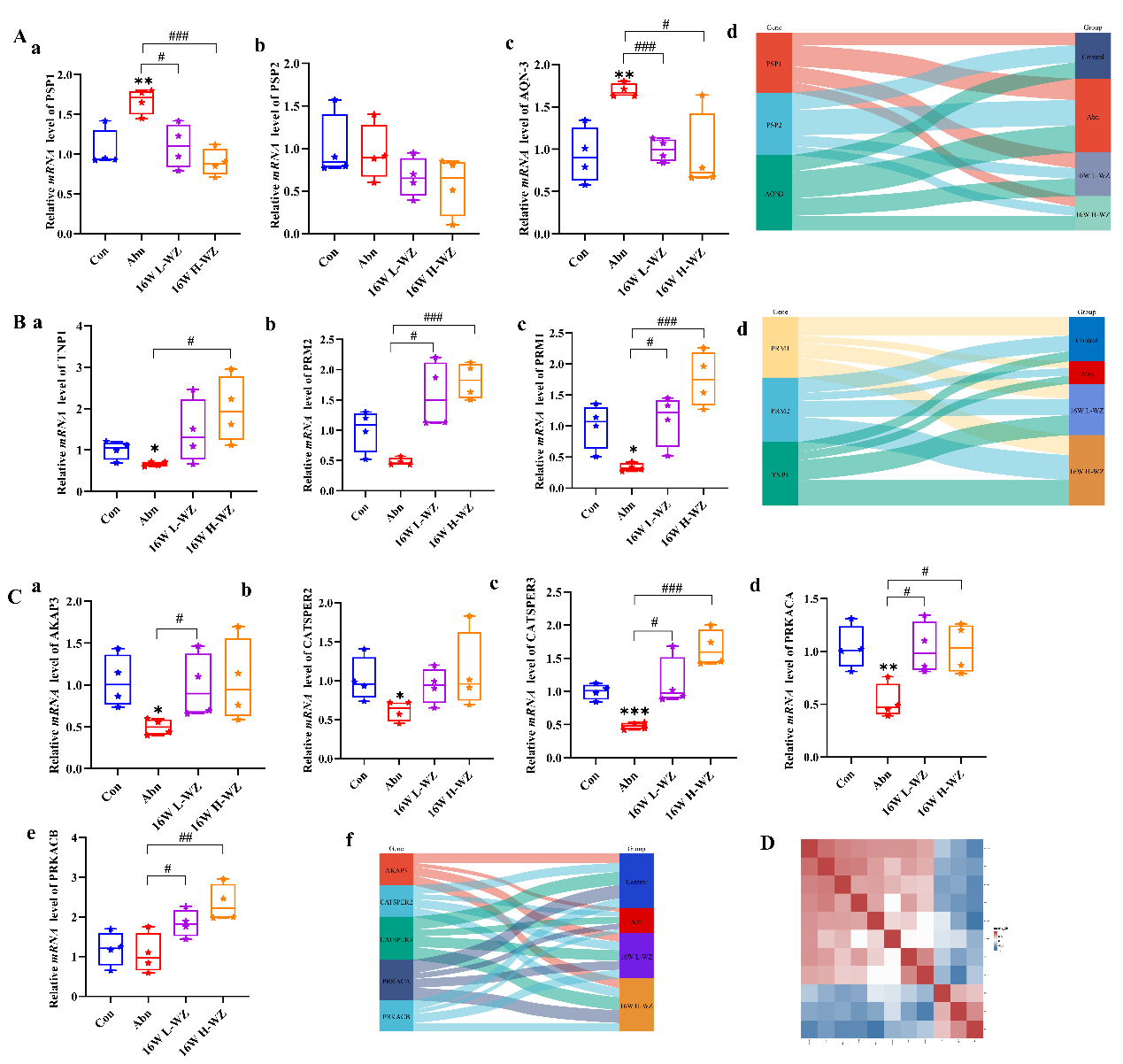
**

**Fig.S2** (A a-d) Sperm adhesion protein family mRNA expression. (B a-d) Sperm chromatin formation-associated mRNA expression. (C a-f) Sperm cAMP-PKA pathway-related mRNA expression. D Heatmap of mRNA correlation. “*” indicated significant difference compared to the corresponding control (*P < 0.05, **P < 0.01 and ***P < 0.001). “#” indicated statistically significant difference between corresponding group (#P < 0.05, ##P < 0.01, and ###P < 0.001)
